# Extracellular matrix proteins and displacement of cultured fibroblasts from duodenal biopsies in celiac patients and controls

**DOI:** 10.1186/1479-5876-11-91

**Published:** 2013-04-08

**Authors:** Leda Roncoroni, Luca Elli, Maria Teresa Bardella, Gianluca Perrucci, Michele Ciulla, Vincenza Lombardo, Carolina Tomba, Dario Conte, Luisa Doneda

**Affiliations:** 1 Center for the Prevention and Diagnosis of Celiac Disease, Gastroenterology Unit II, Fondazione IRCCS Ca’ Granda - Ospedale Maggiore Policlinico and Università degli Studi di Milano, Via F. Sforza 35, Milan, 20122, Italy; 2 Department of Clinical and Community Sciences, Università degli Studi di Milano, Via F. Sforza 35, Milan, 20122, Italy; 3 Department of Biomedical, Surgical and Odontoiatric Sciences, Università degli Studi di Milano, Via Festa del Perdono 7, Milan, 20122, Italy

**Keywords:** Celiac disease, Fibroblasts, Extracellular matrix, Collagen, Tissue transglutaminase, Transglutaminase type 2

## Abstract

**Background:**

Celiac disease (CD) is mainly characterised by villous atrophy and mucosal architectural rearrangement. The fibroblasts (FBs) are the most abundant mesenchymal cell type in the intestinal mucosa and are responsible for both the architectural arrangement of the villi and the formation of the extracellular matrix (ECM). This study aimed at the evaluation of both the intracellular distribution of different proteins involved in ECM and FBs characterisation, and the cellular displacement of primary FBs obtained from duodenal endoscopic biopsies of healthy subjects and celiac patients.

**Methods:**

Primary healthy and celiac duodenal FBs were evaluated by means of immuno-fluorescence assay for collagen type I and IV, fibronectin, actin, alpha-Smooth Muscle Actin (alpha-SMA), Fibroblast Surface Protein (FSP) and transglutaminase type 2 (TG2). The geometric indexes of the fluorescence signals were investigated by image analysis software (Image J, NIH). Both morphology and kinetic were evaluated during a 72 hours time course movie. TG2 medium activity was evaluated by means of ELISA.

**Results:**

All the cells examined were immunopositive for FSP, alpha-SMA, actin, collagen I, collagen IV and TG2. CD cells showed a signet collagen-I and collagen-IV pattern, as compared to the controls being characterised by a spindle geometry. Moreover, the collagen signals in CD FBs showed a significantly higher circularity index (major orthogonal diameter ratio) than the controls (p < 0.0001), whereas the perimeter and area ratio were significantly lower (p < 0.0001). The TG2 signal had a decreased area (p < 0.05), but a two-fold increased medium activity. The time course highlighted a reduction of the displacement of CD FBs.

**Conclusions:**

The isolated primary CD FBs showed a different collagen and TG2 pattern of distribution associated with a different cellular displacement. The reasons for such CD cell peculiar characteristics are yet unknown but they might represent a factor in the progression of the intestinal damage.

## Background

The small bowel mucosal architecture is maintained through a delicate balance among cell production in the crypt compartment, enterocytes migration along the villi, and extrusion of mature epithelial cells from the tip of the villi into the intestinal lumen [1]. This continuous process of cellular differentiation and natural apoptosis arises and is controlled by interactions among epithelium, mesenchymal cells, gut associated lymphoid tissue and the structure of the extracellular matrix (ECM), through mechanisms still largely unknown [2]. Among the different cell types resident in the intestinal mucosa, the fibroblasts (FBs) are the most represented mesenchymal ones and are responsible for both the architectural arrangement of the villi and the formation of the ECM, mainly composed by collagen fibrils [3,4]. As concerns the intestinal wall, ECM (i.e. the structural basis of the human tissues) includes the basement membrane that sustains the epithelium and the villous axis [5]. ECM is mainly formed by fibrillar collagen and provides the support for the different cell types resident in the mucosa (type I collagen) and for the epithelial line of enterocytes forming the intestinal barrier (type IV collagen). ECM also includes the glycoproteins (fibronectin), connecting collagen and cells. All the proteins participating in ECM are interconnected to form a three-dimensional structure by enzymes. The most important of such enzymes is the transglutaminases type 2 (TG2), capable to catalyze stable isopeptide bonds in physiological conditions characterized by neutral pH and low calcium concentrations [6]. Collagen and enzymes are secreted by FBs, which also participate in the tissue re-modelling and repair through their movements along the protein fibrils of the ECM, secreting molecules when and where needed and in association with the other resident cells. Besides this tissutal morphogenetic function FBs actively participate in the inflammatory response by producing chemokines and cytokines, and in the antigen presentation by acting as non-professional antigen presenting cells [7-12]. Moreover, in the autoimmune processes, FBs are frequently targeted by autoantibodies, leading to a biological dysfunction [9,13,14]. Based on the above characteristics, FBs are involved in diseases characterized by tissue architectural alterations, inflammation and autoimmunity.

In the celiac disease (CD), which is the most common autoimmune enteropathy in Western countries, gluten ingestion progressively leads to villous atrophy, crypt hyperplasia with a two to three-fold increased depth of the lamina propria, an increased number of immunological cells (B and T lymphocytes, macrophages and eosinophils) and intraepithelial lymphocytosis [6,15]. These abnormalities, responsible for any functional derangement, are ascribed to the primary cytotoxic effects of gliadin and IgA autoantibodies against TG2 on the surface of FBs and in the ECM [16-18]. In particular, Verbeke et al. reported a derangement of collagen distribution in the duodenal mucosa of CD subjects [16]. As of earlier studies by the Authors [19], duodenal CD FBs can be successfully cultured; they are morphologically different from healthy FBs and potentially carry intrinsic alterations, suggesting their primary involvement in both the CD pathogenesis and the development of the atrophic duodenal lesions.

The present study was aimed at evaluating, in consecutive naïf CD patients and matched controls, the cellular distribution of type I and IV collagens, fibronectin, actin, TG2 and the cellular displacement of primary FBs obtained from duodenal endoscopic biopsies.

## Methods

### Patients

From February 2009 to December 2011, 50 consecutive subjects selected from a cohort of patients undergoing upper GI tract endoscopy for suspected CD, who gave their written informed consent to the study, were enrolled: there were 22 CD patients (10 males and 12 females, mean age 45, range 35–55) and 28 non-CD controls (CTRs) (16 males and 12 females, mean age 42, range 30–60). The CD diagnosis was based on the serological presence of anti-TG2 IgA (ELISA or radio-immunoassay tests) and/or anti-endomysium IgA (immuno-fluorescence technique) auto-antibodies and, at least, a Marsh-Oberhuber grade III duodenal histology. The non-CD group was composed of serologically negative subjects without endoscopic or histological lesions, not reporting other autoimmune or intestinal diseases.

The study plan was approved by the Ethics Committee of the “Fondazione IRCCS Cà Granda-Ospedale Maggiore Policlinico”.

### Duodenal specimens and cell cultures

Three duodenal biopsies obtained from all the participants by standard endoscopic forceps (Boston Scientific, USA) were rapidly dipped into sterile tubes (Becton and Dickinson, Italy) containing an appropriate medium.

The samples were finely chopped with disposable surgery knives and incubated in Ham’s F12 medium (Euroclone, Italy), containing liberase blendzyme 2 (1.4 W/mL) (Roche, Italy) and the cells so derived cultured. Viability was routinely checked by a trypan blue dye exclusion assay (Sigma, Italy) and only those cultures with viability > 95% were used. Mycoplasma contamination was routinely checked and ruled out using the Hoechst method.

### Immunocytochemistry

The different types of proteins were analysed by immunocytochemistry: characterising proteins i.e., the “FB surface protein” (FSP, monoclonal anti-human FSP, Clone 1B10; Sigma, Italy), the α-smooth muscle actin (αSMA) (ABCAM, Italy) and actin (Sigma, Italy), and the ECM components and enzymes i.e., the type I and IV collagens (ABCAM, Italy), fibronectin (Sigma, Italy) and TG2 (Zedira, Germany). Primary antibodies were used following the manufacturer’s recommended dilutions. Cells, seeded onto 24-well plates at a concentration of 20,000 cells/plate, were washed twice 48 hours later in PBS and fixed with 3.7% formaldehyde in PBS for 15 minutes at room temperature (RT). Fixed cells were permeabilised with 0.1% triton X-100 (Sigma, Italy) in PBS for 15 minutes at RT. The non specific binding of any secondary antibody was blocked by incubation with normal foetal serum for 30 minutes at RT. The immunostaining cells were then rinsed with PBS and incubated with a fluorochrome conjugated secondary antibody for 45 minutes at RT, according to the donor species of the primary antibodies. PBS was used as the negative control in place of the primary antibody. Counter-staining was performed using DAPI; the glass coverslip was mounted on glass slides with ProLong Gold Antifade Reagent (Invitrogen, Italy). Images were obtained by fluorescence microscope (Leica, Italy).

The epithelial cellular type was carefully excluded in performing cytokeratine analysis by using anti-human Cytokeratine 20 (Sigma, Italy).

### Medium transglutaminase activity

TG activity was evaluated by a colorimetric technique (Covalab, France) according to the manufacturer’s instructions. Briefly, 50 μL of the cell culture medium was dispensed in each well of the 96-well microtiter plate with covalently coupled CBZ-Gln-Gly and incubated with calcium, dithiothereitol and biotinylated cadaverine; strepavidin-labelled peroxidase was added to the wells and the peroxidase activity revealed using H_2_O_2_ and tetramethyl benzidine. Optical density was measured at 450 nm with a microplate reader (Bouty Diagnostici, Italy). The results were normalized referring to the cell number.

## Image analysis

The digital images were analysed as previously described [19,20]. Briefly, after the localization of fluorescence signals, multiple adjacent high-power fields in each section were selected, acquired, and stored in a personal computer (Power Mac G4, 867 MHz, 640 MB RAM, Apple, USA) in JPEG format (5:1) at 32 bits/pixel on a 764×560 pixel matrix. The stored images were then read by an expert blinded to both image sequence and assignment, and analyzed to determine the differences between CD and CTRs FBs. A 3D interactive surface plot was digitally reconstructed for every image and based on a 256-level scale (0–255).

Fluorescent signal analysis was performed on the digitally elaborated images. Analysis algorithms were developed as a set of macros executed with NIH Image (Interactive 3D Surface Plot Plugin), which is an integrated image-processing software distributed as freeware by the National Institutes of Health (Bethesda, USA). Prior to analysis an automated threshold process was performed on the images to minimize the influence of light variation in the microscope field and in the operator’s subjective settings. This process cuts off any object below the minimum signal intensity. FBs were recognized on the basis of their size and intensity signal using a cell count algorithm which draws a region of interest (ROI) around each discrete object within the image. The minimum size in pixels of the objects to be included in the count was previously defined by accurately measuring twelve representative FBs. Objects below the minimum size were not included in the analysis. The evaluation of the fluorescent signal included, on one hand, the major orthogonal diameters and their ratio as index of circularity and, on the other hand, the perimeter, the area and their ratio, as index of complexity.

### Shape and motility evaluation

Morphology and growth were evaluated during a 72-hour time course movie. Images were acquired with a Leica DMI AF6000LX microscope at 10× magnification.

After three days from seeding, the cell culture plates (7 CD and 7 CTRs) were placed under the microscope connected to a camera. The camera was set for capturing frames every 10 minutes over 72 hours. The compressed frames were composed into the experimental movies (one for each plate); the movies were accelerated by software (LAS-AF imaging software), obtaining a 40 seconds long one. Thus, one second of the final digital movie corresponded to a 1.8 hours long period of culture. Fifteen randomly selected cells from 3 different movies from each group were selected at time 0 (i.e. at the beginning of the movie). The nucleus was fixed to track the displacement after 12, 24, 36, 48, 60 and 72 hours (Photoshop Adobe, USA). Data were expressed as total displacement (μm) and velocity (μm/h). Also perimeter, circularity index and feret diameters were analysed as previously shown at time 0 and after 36 and 72 hours.

## Statistical analysis

Data were expressed as mean ± standard deviation (SD) or median and range. Comparisons of the parameters were carried out by one-way ANOVA. Statistical analysis was performed using statistical software (SPSS version 13, SPSS, USA), considering a p value < 0.05 as significant.

## Results

**Figure 1:**
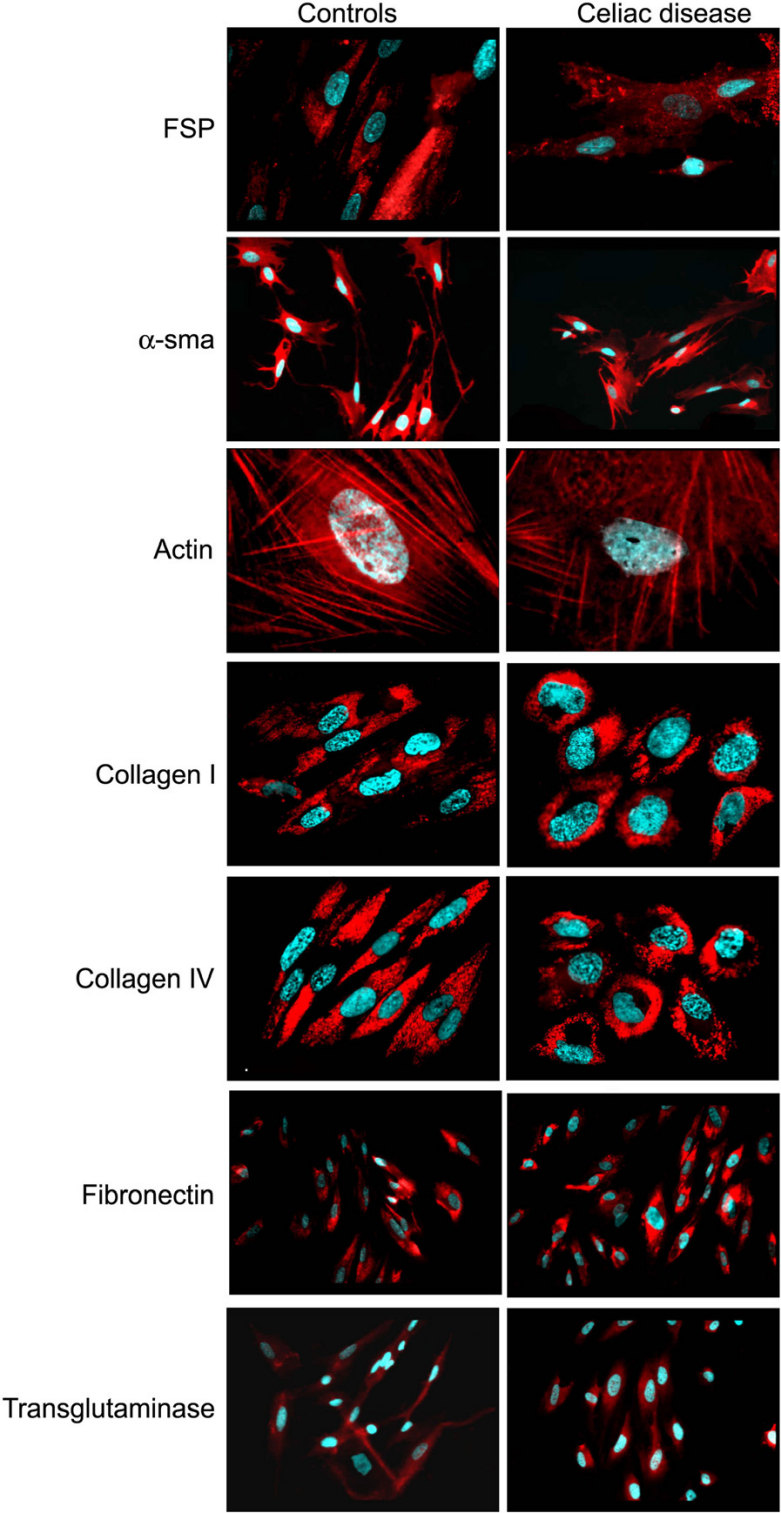
Fibroblast surface protein, α-smooth muscle actin, actin, type I and IV collagens, fibronectin and transglutaminase type 2 immuno-fluorescence analysis of fibroblasts from celiacs and healthy controls.

**Figure 2:**
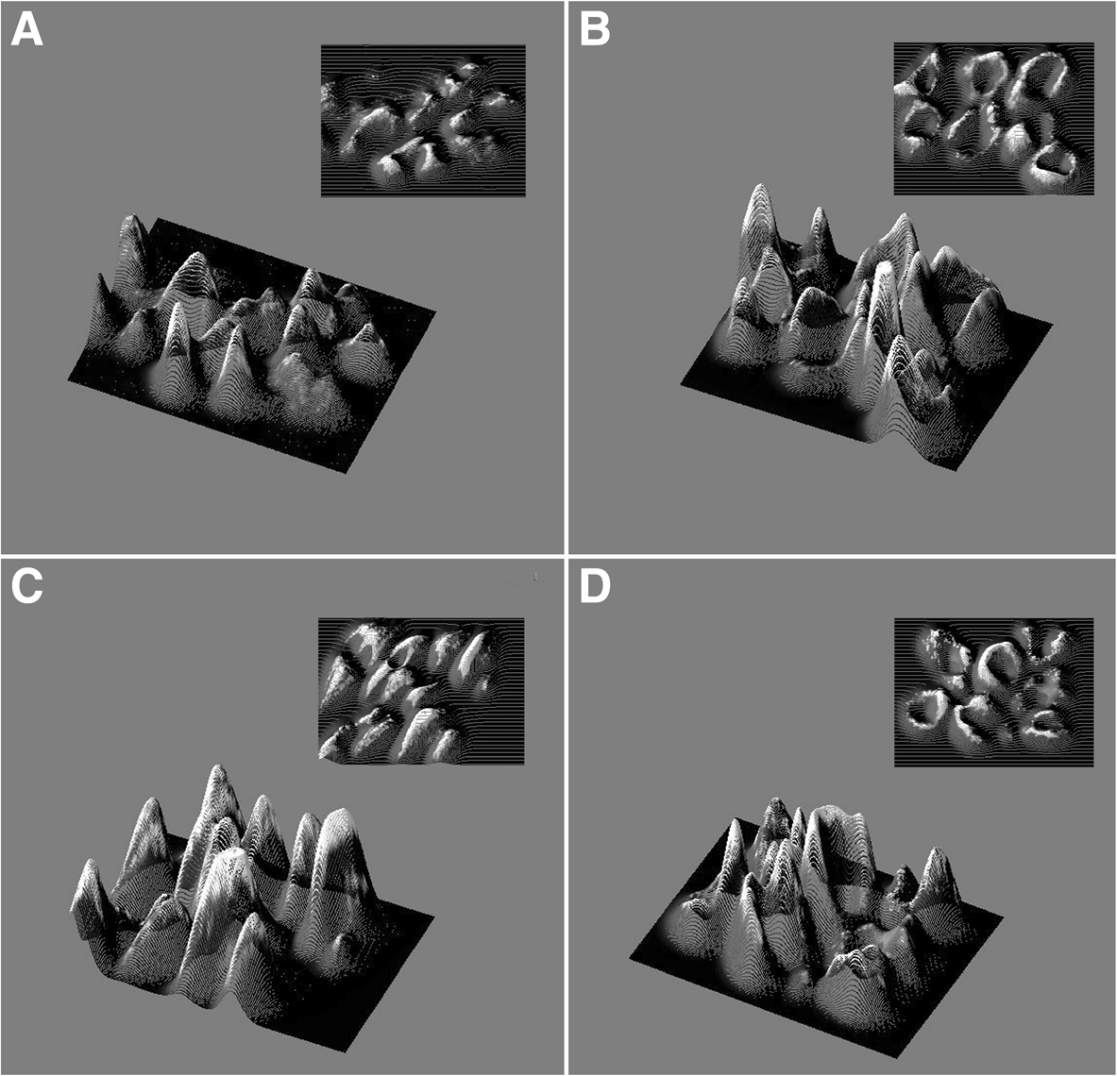
Digitally reconstructed images from immunofluorescence analysis of type I and type IV collagen of fibroblasts from healthy controls (panels A and C) and celiacs (panels B and D), respectively.

**Figure 3:**
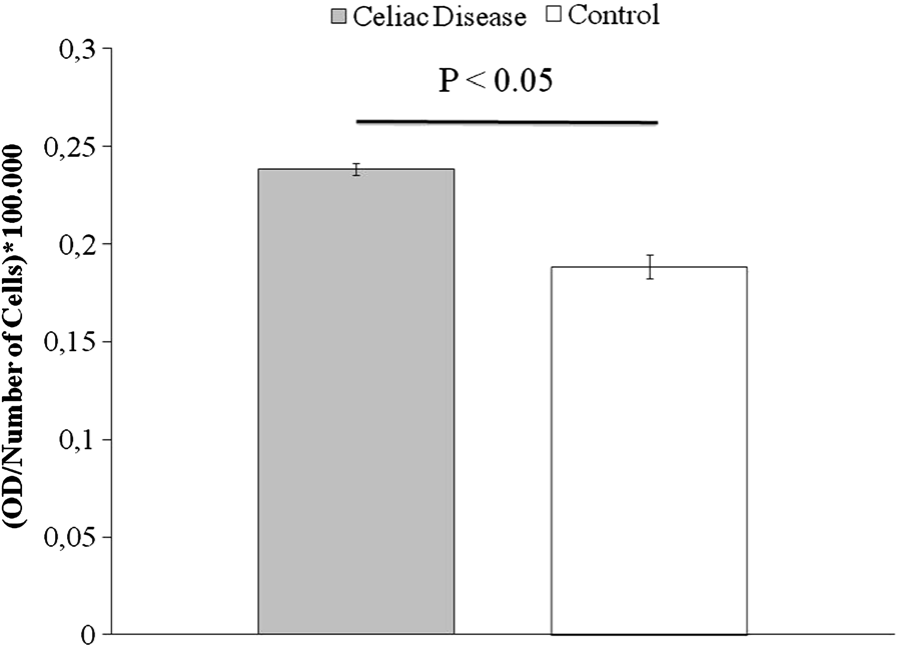
Transglutaminase activity in the culture medium of fibroblasts from celiacs and healthy controls.

Both CD and CTR cells expressed FSP, α-SMA, actin, type I and IV collagens, fibronectin and TG2 (Figure 1) but not cytokeratine 20. CD cells showed a signet type I and IV collagen patterns, whereas CTRs had a spindle geometry (Figure 1). Type I and IV collagen morphometric analysis, performed on CD and CTR FBs obtained on the same day of culture, showed some differences. As detailed in Table 1 and Figure 2, which shows the digitally elaborated images, the collagen fluorescent signal involved a larger part of the FB cytoplasm with an increased circularity index in CD FBs compared to CTR ones. Statistically significant differences between the TG2 fluorescent signal of CD and CTR FBs were found. In particular, in the CD FBs the area covered by the TG2 fluorescent signal decreased by 50% (Table 2), while TG activity increased in the culture medium of CD FBs (Figure 3). No statistically different findings were observed for αSMA, actin and fibronectin signals (data not shown). The cellular shapes of CD and CTR FBs are detailed in Table 3.

**Table 1:** Morphometrical characteristics of intracellular fluorescent signal of type I and IV collagen fibroblasts from celiacs and healthy controls

	**Type I collagen**			**Type IV collagen**		
**Parameter**	**Controls (#12)**	**Celiacs (#12)**	**P value**	**Controls (#12)**	**Celiacs (#12)**	**P value**
Feret Diameter (μm) *	53.00 ± 11.00	35.20 ± 5.00	< 0.005	62.90 ± 12.05	32.90 ± 4.90	< 0.0001
Perimeter (μm)	121.30 ± 24.00	107.60 ± 16.80	< 0.005	145.90 ± 27.20	99.60 ± 12.06	< 0.001
Area (μm^2^)	377.20 ± 86.90	563.40 ± 122.80	< 0.05	375.60 ± 118.60	502.70 ± 98.10	< 0.05
Circularity index°	0.30 ± 0.10	0.62 ± 0.11	< 0.005	0.20 ± 0.06	0.60 ± 0.11	< 0.0001
Complexity index (1/μm)^&^	0.30 ± 0.03	0.19 ± 0.02	< 0.0001	0.40 ± 0.08	0.20 ± 0.03	< 0.0001

* Feret diameter: longest axis length.

° Circularity index: Feret diameter/short axis length.

^&^ Complexity index: perimeter/area (1/μm).

**Table 2:** Morphometrical characteristics of the intracellular fluorescent signal of the transglutaminase type 2 in fibroblasts from celiacs and healthy controls

**Parameter**	**Controls (#12)**	**Celiacs (#12)**	**P value**
Feret Diameter (μm)*	26.60 ± 5.40	19.10 ± 4.40	<0.05
Perimeter (μm)	73.00 ± 23.10	53.10 ± 13.80	NS
Area (μm^2^)	143.90 ± 50.70	90.00 ± 34.80	<0.05
Circularity index°	0.38 ± 0.20	0.40 ± 0.10	NS
Complexity index (1/μm)^&^	0.50 ± 0.10	0.60 ± 0.20	NS

* Feret diameter: longest axis length.

° Circularity index: Feret diameter/short axis length.

^&^ Complexity index: perimeter/area (1/μm); *NS,* Not significant.

**Table 3:** Morphometrical characteristics of fibroblasts from celiacs and healthy controls

	**Characteristics of fibroblasts**		
**Parameter**	**Controls (#12)**	**Celiacs (#12)**	**P value**
Feret Diameter (μm)*	62.90 ± 21.60	89.00 ± 28.00	< 0.05
Perimeter (μm)	188.30 ± 61.90	252.30 ± 99.80	< 0.05
Area (μm^2^)	744.60 ± 357.60	933.60 ± 649.30	NS
Circularity index°	0.30 ± 0.20	0.20 ± 0.10	NS
Complexity index (1/μm)^&^	0.30 ± 0.10	0.40 ± 0.20	NS

* Feret diameter: longest axis length.

° Circularity index: Feret diameter/short axis length.

^&^ Complexity index: perimeter/area (1/μm); *NS*, Not significant.

**Figure 4:**
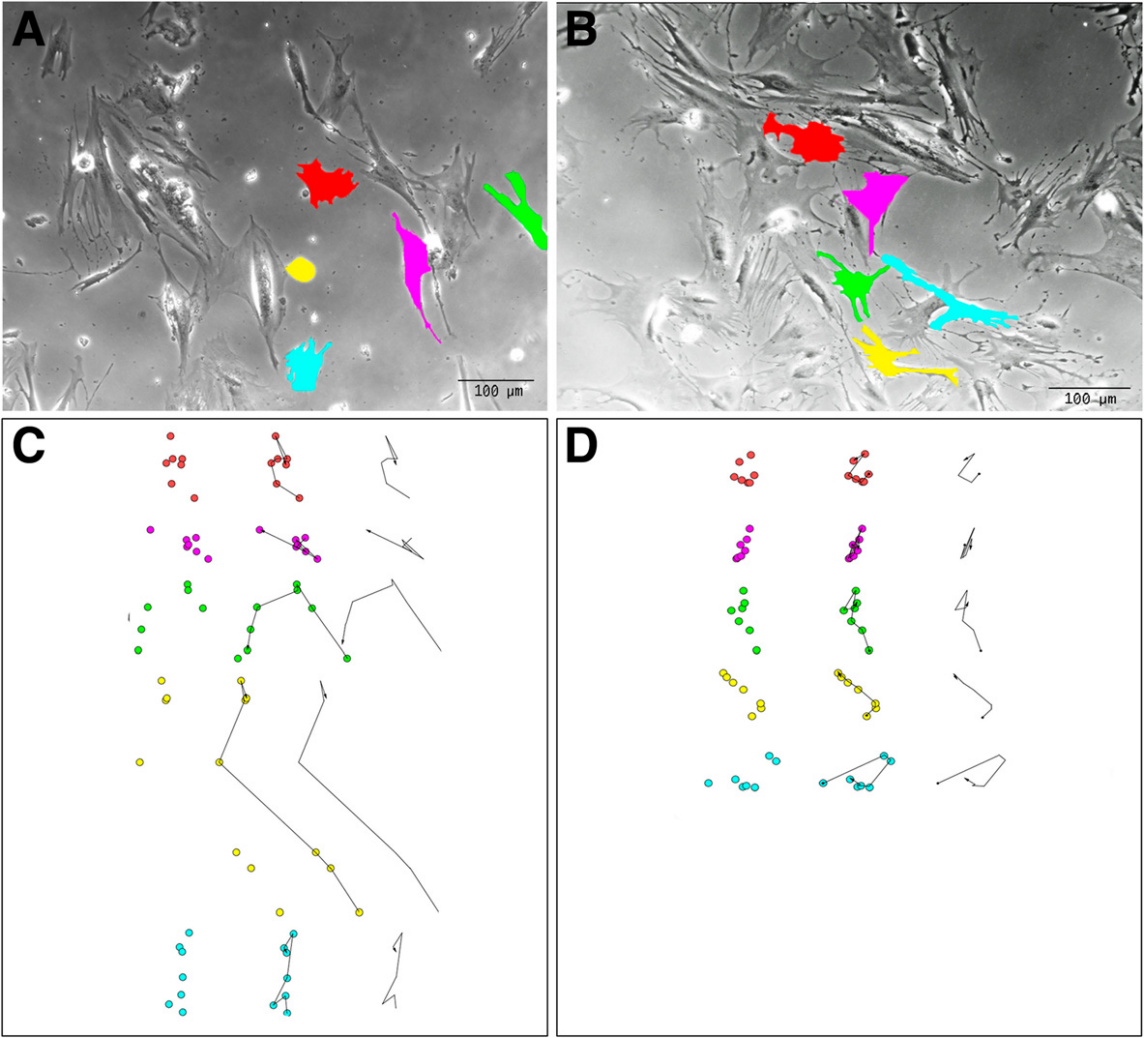
**Motility tracking of fibroblasts from healthy controls (panel A) and celiacs (panel B).** In the upper panels the images at the beginning of the movie recording are reported with the cells tracked. In the lower panels the movement of the cell nuclei into the field after 12, 24, 36, 48, 60 and 72 hours are reported with the final trace.

The CD FBs showed a decreased motility and velocity. In detail, during the 72 hours period of the observed culture the CD FBs moved 113 ± 39 μm while the CTR FBs 222 ± 88 μm with a velocity of 1.5 ± 0.5 μm/h *vs* 3.0 ± 1.2 μm/h, the difference being statistically different (see Figure 4 for movement tracking). Two additional movie files show the FBs displacement in more detail (Additional file 1: Video S1 and Additional file 2: Video S2 for CD and CTR FBs respectively).

## Discussion

The results from the present study are consistent with an alteration of the collagen and TG2 immunofluorescence signal in the CD FBs compared to the CTR ones. These abnormalities could be involved in the decreased motility observed during the time-course experiments and in turn in the villous damage observed in CD.

The maintenance of a normal duodenal mucosal morphology results from a continuous interaction between the epithelium, the ECM represented by the basement membrane and the underlying FBs network, responsible for the secretion of collagen molecules and matrix stabilizing enzymes (TG2) [6]. In CD the mucosal structure is deeply altered as proven by the typical “stigmata” of CD represented by mucosal atrophy, crypt hyperplasia and lymphocytic infiltration. Also the lamina propria, composed by type IV collagen and controlling the interface between the epithelium and the subepithelial compartment, is altered in CD [21,22]. These aforementioned ECM alterations can be attributed to the secreted autoantibodies (anti-TG2 and anti-gliadin) interacting with the matrix proteins or alternatively to a primary defect of CD FBs which could abnormally express these proteins or enzymes [23]. Moreover, FBs control the degradation of ECM through the secretion of lytic enzymes, as the metalloproteases (MMP), and their inhibitors [24-26]. Previous findings suggest that both these mechanisms can play a role in the CD progress. Schuppan et al. for example have demonstrated that ECM could act as a reservoir of autoantibodies, fuelling the mucosal inflammation and eventually modifying the pH and calcium concentrations, making the environment suitable for protein lysis and TG2 activation in terms of switching from isopeptide bonds to the deamidation using H_2_O as acyl acceptor [6,23]. On the other hand, Verbeke et al. have reported a decreased immunofluorescence signal of type IV collagen and laminin with a “leaky” basement membrane [16]. Accordingly, the increase of type I and IV collagen signals in FBs and the increase of TG2 activity in the medium could represent an attempt of CD FBs to restore the decreased collagen levels in the ECM of CD patients and to increase the formation of isopeptide bonds stabilizing the matrix fibrils. Although demonstrated only *in vitro* this scenario could also be present in the flattened CD mucosa; in other words, as primary cell cultures maintain the memory of their site of origin even after different passages, a restoration of the original *in vivo* scenario could be possible [27,28]. To underline these mechanisms, the fibronectin levels did not differ between CD and healthy FBs, in line with the results obtained by Korhonen et al. who reported a comparable pattern both in CD and non-CD duodenal mucosa [29].

In the present study FBs resulted positive for αSMA, which is the most relevant marker of myofibroblasts (mFBs) and represents an intermediate state between FBs and smooth muscle cells [30]. As observed in our experiments, mFBs display prominent cytoplasmatic actin microfilaments (stress fibers) and are connected to one another by adherent and gap junctions [10]. In the gastrointestinal tract two kind of mFBs are usually recognized, the α-SMA positive and those negative, resident in the apical and basal parts of the villi, respectively [30]. In detail, α-SMA positive FBs correspond to our *in vitro* population and are involved in both epithelial differentiation and ECM formation [12]. More recently, using immunohistochemistry, some authors have demonstrated that α-SMA positive subepithelial mFBs constitutively express class II MHC molecules and are distinct from professional antigen presenting cells [31].

A further interesting finding from the present series, reported for the first time, is represented by the decreased motility of CD FBs compared with the control ones, as indicated by a 50% reduction of the displacement of CD FBs during the 72-hour movie recording. This finding refers to different biological aspects, as cell shape/dimension and migration are dynamic processes connected to the cellular fate (growth/death) and depending on the ECM composition and endocellular cytoskeleton. Thus, round shapen cells (circularity index = 1) appear as in stationary state while a Y-like shape as frequently observed in FBs cultures (circularity index < 1) characterizes those cells ready for migration [32]. In fact, the “Y” shape is the most favorable for polarized migration, with a frontal adhesion point and a rear arc where active forces as given by the Laplace law on tension, prepare for the movement of the cell [32]. The molecular mechanisms underlying cellular motility are largely unknown, even if the Rho family of GTPases seems to play a crucial role [33]. Beyond the complex molecular processes connected to FB motility, several relevant clinical consequences have been reported. In particular, decreased FB motility has been reported in patients affected by diabetes mellitus (DM): for such patients a migratory defect represents a relevant impairment for wound healing, responsible for chronic skin ulcerations even requiring amputation [34]. The discussed finding could represent a connection between CD and DM, which are supposed to present some common factors such as genetic background. Moreover, DM animal models are currently used to investigate particular aspects of CD as they are capable to develop duodenal atrophy when exposed to gluten feeding [35]. It remains an interesting point for future investigation whether the observed alterations are common to other gastrointestinal autoimmune disorders (inflammatory bowel disease, autoimmune enteropathy) or they could be reverted in case of FBs derived from CD non atrophic mucosa, where a different FB population has been selected from duodenal micro-environment.

## Conclusion

Overall, the results from the present study highlight the importance of FBs in CD pathogenesis, strongly supporting the need for further investigations on this topic in order to improve our understanding of the CD related injury processes. Transitionally, if the defect of tissue repair process in CD FBs should be confirmed as pivotal in the normalization of duodenal mucosa after gluten withdrawal from a patient’s diet, the possibility of ECM targeted therapies could play a role in the CD research.

## Competing interests

The authors declare that they have no competing interests.

## Authors’ contributions

LR and VL perfomed the cell cultures, immunofluorescence eperiments and ELISA. GP and MC dealt with the FB movies and image analysis. LE and CT planned the study, dealt with the patients and performed upper endoscopies with duodenal biopsies. MTB, DC and LD analyzed data and prepared the manuscript. All the authors read and approved the final manuscript.

## Supplementary Material

Additional file 1: Video S1 CD FBs. Click here for file

Additional file 2: Video S2 CTR FBs. Click here for file
